# Social Media and Attitude Change: Information Booming Promote or Resist Persuasion?

**DOI:** 10.3389/fpsyg.2021.596071

**Published:** 2021-06-24

**Authors:** Yizhi Wang, Yuwan Dai, Hao Li, Lili Song

**Affiliations:** ^1^Department of Psychology and Beijing Key Laboratory of Behavior and Mental Health, Peking University, Beijing, China; ^2^School of Psychological and Cognitive Sciences, Peking University, Beijing, China; ^3^Plateau Center of Brain Sciences, Tibet University, Lhasa, China; ^4^School of Medicine, Tibet University, Lhasa, China; ^5^CAS Key Laboratory of Mental Health, Institute of Psychology, Beijing, China; ^6^Department of Psychology, University of Chinese Academy of Sciences, Beijing, China

**Keywords:** attitude change, social media, information overload, Weibo, repeated information, persuasion

## Abstract

Emerging social media platforms such as Twitter and its Chinese equivalent Weibo have become important in information-sharing and communication. They are also gradually becoming stronger in guiding public opinion. When compared with traditional media, these platforms have salient characteristics, such as highly efficient dissemination of information and interactive commentary, which can contribute to information overload. In earlier research, only the effect of social media on attitude change has been studied, but the specific mechanism of this effect in the context of information overload has not been found. To answer this question, we measured the attitude change of participants after they read Weibo posts about street vendors. A 2 (post-attitude: positive posts vs. negative posts) × 4 (reading time: 35 vs. 25 vs. 15 vs. 5 min) experiment was set up, and the Single Category Implicit Attitude Test was used to measure the implicit attitudes. The interaction effect revealed that in both positive and negative posts, less reading time (i.e., information overload) had a stronger influence. Users were more easily persuaded by posts under high overload. Furthermore, the changes in the attitudes of users were not simply stronger with more information. We found three stages, namely, obedience, resistance, and acceptance, with different mechanisms. Therefore, in the positive information overload condition, the attitudes of individuals eventually change in a positive way. In the negative information overload condition, individuals tend to be biased against the group being reported.

## Introduction

In our daily life, small preferences such as specific brands of hotel, or political behaviors such as voting, are the ways that we express our attitudes. Attitude change and persuasion are the core concepts in studying individual behavior (Crano and Prislin, 2006). Companies invest a lot to change the attitudes of customers (Alba and Hutchinson, 2000). Similarly, people also use various persuasion strategies to convince others (Briñol and Petty, 2010). Announcements on mainstream media such as television and newspapers are one of the most effective persuasion methods, thus attracting the attention of researchers. However, social media platforms, such as Twitter and Weibo (its Chinese equivalent), have gradually become invaluable platforms for online social interactions (Parslow, 2011). They can casually change the impressions of specific groups. For example, a positive voice on social media platforms has caused environmentalists to obtain attention and support within a short time (Herron, 2015). However, over time, the platforms have been overwhelmingly criticized and accused of ignoring economic development issues and causing the retrogression of social civilization (Chung and Cho, 2017). Evidently, the new social media platforms are more diverse and thus could change the attitudes of audiences more rapidly than traditional media (Lee and Ma, 2012).

The use of these platforms for attitude change has already taken effect in the business community. They have shaped public opinion and are more appealing, especially as direct tools of online advertising and shopping (Lee and Lee, 2004). According to the China Internet Network Information Center (2019), the number of Weibo users had risen to 337 million by June 2018. Most people realize that social media posts potentially change various aspects of the attitudes of people, such as contributing to market products (Jansen et al., 2010), maintaining brand reputation (Becker et al., 2013), and assisting enterprises to recover after a crisis (Van Norel et al., 2014). In addition, there are other online factors that affect the attitudes of people, such as emotion (Hudson et al., 2015), motivations (Taylor et al., 2020), and so on.

However, individuals tend to maintain a stable attitude rather than one that shifts easily. People use different strategies, such as attitude bolstering, counter-arguing, negative affect, selective exposure, social validation, and source derogation, to resist attitude change (Jacks and Cameron, 2003). Some even tamper with their own memories to remain consistent with their initial attitude (Ross et al., 1981). Besides, people also select evidence from new information, interpret it, and make it consistent with their original beliefs and attitude (Albarracín and Mitchell, 2004). In cases where the new information does not match the previous attitude, they retrieve the old context from their long-term memories (Briñol et al., 2004). Similarly, people tend to seek information from memory to strengthen and maintain their original attitudes and beliefs. There is always an inhibitory defense in attitude change, which causes people to keep their previous attitude in cases where the new information is not convincing enough. Too much pressure from new information further causes individuals to resist persuasion and become more certain of their initial attitude (Tormala and Petty, 2002). However, why do social media have such a great influence on public opinion?

One of the salient features of social media is that everyone can be a source of information, unlike in traditional media where the source is often authoritative after screening and verification (Hayes and King, 2014). However, anyone can publish a post on social media at any time; usually, neither screened nor verified. In addition, public opinion associated with these posts begins to build up. In most cases, some users blindly follow and support opinion leaders, thereby posting new content that further exacerbates the trend of public opinion (Forelle et al., 2015). Public opinion sometimes has a certain degree of blindness regarding social media content, thus causing people not to trust it as much as mainstream media. Evidence from the 2018 Edelman Trust Barometer Special Report concluded that only 41% of people say they trust social media. Cognizant of this, people spend more time and energy to discern whether the information is worth believing, thereby causing more obstacles to cognitive processing. The duplication of publishers of information in social media platforms requires more cognitive resources.

Moreover, social media platforms have a large selection set. They are totally different from the simple one-after-another mode in the old media. As such, users can be inundated with hundreds or even thousands of posts from various posters in minutes (Zhang L. et al., 2016). The processing of this information leads to challenges such as uncertainty, diversity, ambiguity, novelty, and complexity. This further leads to cognitive overload, thereby affecting the understanding and interpretation of the information (Eppler and Mengis, 2010; Paas et al., 2010). A recent study revealed that using social media platforms unwittingly increases cognitive overload and this further weakens understanding, especially in reading tasks (Jiang et al., 2016). One of the factors leading to individual cognitive load is information overload, which is the psychological state in which an individual subjectively perceives that the amount of information he/she receives exceeds his/her information processing ability (Ferrari, 2010). That is, the brain has insufficient cognitive resources. At present, the Internet has become the primary cause of information overload in our society. The amount of digitized information is increasing rapidly, and its types are more varied (Schultz and Vandenbosch, 1998). Moreover, the quality of online information is uneven. Thus, it is hard to distinguish between true and false information. At the same time, the number of information noise, irrelevant information, untruthful information, ambiguous information, and alternative options increases (Eppler and Mengis, 2004; van Knippenberg et al., 2015; Zhang S. et al., 2016), which may negatively affect the mental or physical health of individuals (Matthes et al., 2019). The information overload of social networking sites will further lead to the negative social comparison and social fatigue of users (Lee et al., 2016; Fu et al., 2020; Niu et al., 2020). Information overload can also cause consumers to make worse decisions when they shop online (Chen et al., 2009). In addition, information overload can reduce the productivity and creativity of employees, which has a negative impact on society (Fu et al., 2020).

Given that the two inherent features of social media platforms, namely, the explosive dissemination of posts and the many indistinguishable voices, raise new problems, the voices on social media require more cognitive resources for processing. Once the information load exceeds the processing limit, it causes problems of resource allocation and affects the efficiency of problem-solving, thereby leading to cognitive overload (Chernev et al., 2010). During this time of insufficiency in cognitive resources, it becomes more difficult to search for evidence in memory to strengthen the original attitude. Thus, processing and understanding of the new information are the only aspects left in consciousness (Greifeneder et al., 2010). For this reason, people are unable to process new information deeply, thereby obtaining only a low level of information due to the limited cognitive resources (Inbar et al., 2011). Since people do not focus on the logic and evidence of the information, they are more easily influenced. In this study, cognitive overload may play an important role that underlies the conspicuous attitude changes while reading social media posts. We tested this question using Weibo as a platform and used an Implicit Attitude Test (IAT) to assess attitude changes. We expected that a high cognitive overload would be more persuasive than a low cognitive overload. In addition, we predicted that participants would not be directly persuaded and there would be an attitude defense.

## Materials and Methods

Informed consent was given by each participant before experiments. The experiments were in accordance with the Declaration of Helsinki and approved by the Ethics Committee of the Department of Psychology, Peking University.

To exclude the interference of personal preferences or values, we used the vendors as the comment group. Moreover, people generally hold a neutral attitude toward vendors.

### Cognitive Overload Manipulation

Limited reading time was used to manipulate cognitive overload. When time is limited, participants have less time to examine and process the information. Participants would read 18 Weibo posts. Each post is about 100 words, and 25 min is a suitable time to read all the posts, which was tested by two research assistants (*M* = 25.43, *SD* = 1.46). Participants would be asked to finish reading all the posts in 35, 25, 15, and 5 min. A shorter time imposed a higher level of overload.

### Positive/Negative Post-attitude

Participants would read 18 Weibo posts. On the positive post-attitude condition, participants would read 12 positive posts about vendors and six unrelated posts. Two positive posts were presented, then one irrelevant post was presented, and this order was repeated. Similarly, on the negative post-attitude condition, participants would read 12 negative posts about vendors and six unrelated posts. Two negative posts were presented, then one irrelevant post was presented, and this order was repeated. All the positive/negative posts were searched by research assistants using words such as “street vendor” and “vendor” on Weibo. The six unrelated posts were selected from popular posts on Weibo and rechecked if all of them were unrelated to vendors.

### Term Rating

Three postgraduate students in psychology found 22 terms (i.e., vendors, stall owners, small traders, street peddler, small bosses, small shops, practice stalls, stall owners, shopkeepers, traders, small retailer, peddler, packman, supermarket owners, booth-keeper, small businessmen, small shop owners, the vendors selling fruits, the vendors selling drinks, the vendors selling vegetables, the vendors selling medicines, and the vendors selling groceries), 24 positive adjectives, and 24 negative adjectives. We collected 100 positive words and 100 negative words associated with “street vendor,” a total of 200 words, such as good-mannered, single-hearted, stingy, and self-contemptuous. Then, 30 graduate students used the seven-point Likert scale (“1” means “the most unrepresentative” and “7” means “the most representative”) to assess the extent to which the above-described original attribute words represented positive and negative descriptions of the “street vendor.” In this way, we obtained a total of 48 positive and negative words with 24 in each category from an Internet search to define street vendors, and all the items used are listed in Table 1. In the term rating task, 120 participants from Peking University (*M* = 21.31, *SD* = 2.69 years) were then asked to perform a simple rating task. The term rating task consisted of two parts, namely, definition term rating and adjective term rating. They did not take part in the experiment of the Single Category Implicit Attitude Test (SC-IAT). First, they did definition term rating and determined whether the noun terms in the materials were associated with street vendors. Each participant was given the following definition of a street vendor:

*A street vendor is a person who offers goods or services for sale to the public without having a permanently built structure but rather with a temporary static structure or mobile stall. They could be t-shirt vendors, street artists, fancy food trucks among others*.

**Table 1:** All adjective items of “street vendors” and rating scores (*N* = 120).

Positive				Negative			
Terms	*M*(*SD*)	Terms	*M*(*SD*)	Terms	*M*(*SD*)	Terms	*M*(*SD*)
Good-mannered	6.91(0.33)	Popular	6.25(0.79)	Stingy	6.73(0.26)	Hidebound	6.38(1.42)
Single-hearted	6.85(0.65)	Responsible	6.22(1.04)	Self-contemptuous	6.72(0.65)	Bad-mannered	6.35(1.26)
Trustworthy	6.83(0.66)	Kind	6.17(0.63)	Grasping	6.71(0.83)	Unlucky	6.27(1.04)
Committed	6.79(0.59)	Courageous	6.15(1.25)	Irritable	6.70(1.19)	Depressed	6.21(1.39)
Personable	6.64(1.03)	Clean	6.10(1.02)	Insincere	6.68(0.43)	Morbid	6.15(0.46)
Talented	6.60(1.24)	Steady	6.06(1.33)	Insidious	6.66(0.54)	Unamiable	6.10(0.45)
Capable	6.58(0.76)	Excellent	6.03(0.49)	Underbred	6.65(0.94)	Naive	6.05(1.23)
Caring	6.54(0.42)	Honest	6.01(1.33)	Terrible	6.62(1.24)	Illiterate	5.87(0.79)
Righteous	6.46(1.12)	Efficient	5.96(0.94)	No-potential	6.61(1.04)	Spiritless	5.79(1.29)
Captivating	6.45(0.98)	Earnest	5.83(0.48)	Distrustful	6.53(0.55)	Corruption	5.73(0.82)
Persevering	6.38(0.87)	Unselfish	5.79(0.77)	Shameless	6.47(0.36)	Uncouth	5.60(1.02)
Potential	6.26(0.54)	Gregarious	5.53(1.22)	Evil	6.40(1.11)	Vulgar	5.57(0.68)

Each participant was then asked to indicate whether each term was likely to be related to street vendors by clicking on-screen buttons labeled “yes” and “no.” A “not sure” selection was also available for cases where the participant was not familiar with the search term.

Then, the participant performed another rating task to determine whether the given adjectives were positive or negative when used to describe street vendors. Each participant was first given the adjective list and asked to make a choice by clicking on-screen buttons labeled “positive” and “negative.” The participant was also able to select “not sure” if he or she was not sure about it. All the items used are listed in Table 1.

The participants were finally asked to determine whether posts about “street vendors” on the social media platform were positive or negative. The posts were selected on Weibo using words such as street vendors and similar terms were noted earlier.

In each part, all terms were presented on the screen individually in random order. Each participant was paid US$2.50. They were finally selected based on the rating scores.

### Single Category Implicit Attitude Test

The Implicit Attitude Test (IAT) is widely used to measure explicit attitude. When compared with traditional self-report measure, the IAT is distinct and effective: it is less likely to be influenced by social approvability, expectations, or subjective factors, and it can effectively measure attitudes that people are aware of but do not want to report their attitude change about a certain group. Besides, when compared with other implicit attitude measures, IAT is also sensitive to catch subtle changes (Greenwald et al., 2003). IAT fits well with our experimental design, which measures the attitudes of people and prejudices toward street vendors. In this study, we chose SC-IAT to measure explicit attitude.

The SC-IAT (Karpinski and Steinman, 2006) measures the strength of association between a single target concept and evaluative attributes by reaction times. It is based on the same principles as the IAT but with a single object. In the first phase, the “positive” category was sorted with one key (on the left, e.g., the “a” of a keyboard) while the “negative” category and the target concept were sorted with another key (on the right, e.g., the “p”). In the second phase, the target concept was no longer associated with the “negative” category but with the “positive” category and thus required the use of the other key. The respondent had to press one of the two keys as quickly as possible to categorize stimuli (i.e., target concept with either a positive or negative word) that appeared at the center of the screen. A strong association in memory of a participant had a short reaction time while a weaker association had a longer reaction time. The difference in reaction time between the two combinations (after several transformations, i.e., the D-score algorithm; Greenwald et al., 2003) thus reflected the attitudes of individuals regarding the target object. In this study, the SC-IAT was created using the “street vendor” definitions and adjectives obtained after rating.

### Participants

The G∗Power 3.1.9.7 program was used to estimate the number of samples required for the study (Faul et al., 2007). Using a medium effect size *f* = 0.25, power = 0.95, and two measurement indicators, the detection of significant inter-group interaction effects in the multivariate ANOVA required a minimum of 220 participants in groups of 25 per test. The participants were students from Shanxi Normal University. All participants used social media platforms in daily life. They did not have family or relatives who were street vendors. We excluded participants who did not pass the test questions designed to check whether they had read the materials carefully. The remaining participants were 119 males (*M* = 22.96, *SD* = 2.87 years) and 121 females (*M* = 22.40, *SD* = 2.79 years).

### Study Procedure

This study was conducted in accordance with the ethical standards of the American Psychiatric Association. It was made known to the participants that they were free to participate or not. They were also notified that their responses were anonymous. They also had the liberty to withdraw from the study at any time.

Participants worked on several tasks. First, they were instructed to read 18 Weibo posts. On the positive post-attitude condition, the materials comprised 12 positive posts and six unrelated posts. On the negative post-attitude condition, the materials comprised 12 negative posts and six unrelated posts. The time-limits to finish reading were 35, 25, 15, or 5 min (Qiu-mei, 2015). Then, they responded to three check questions about the posts, such as “There is a Weibo telling that one animal left Chernobyl forbidden zone. Please specify the kind of animal? A. Dog B. Fox C. Wolf.” Participants fail greater or equal to two questions would be evaluated not read the posts carefully and be excluded.

The participants then completed the SC-IAT in two phases, namely, street vendor definition items plus positive adjective items vs. negative items, and street vendor definition items plus negative adjective items vs. positive items. Each phase included practice periods. Each practice period comprised 24 trials and was followed by a measurement phase of 72 trials. The instructions were similar to those given for the IAT. Prior to each test, a member of staff ascertained that the participant had understood the instructions. Feedback was given for the action of each participant. On one hand, when a participant pressed the wrong key, a red “X” appeared in the center of the screen for 150 ms and was followed by the next trial. On the other hand, when a participant gave a correct response, a green “O” appeared for 150 ms. If none of the keys was pressed within 1,500 ms, the message “Please faster” appeared in the middle of the screen. The participants also provided general demographic information and were then thanked, paid, and debriefed (Table 2).

**Table 2:** The procedure of “SC-IAT.”

Block	Trails	Task	Response	
			“F” key	“J” key
1	24	Practice	Positive adjective + “street vendor”	Negative adjective
2	72	Test	Positive adjective + “street vendor”	Negative adjective
3	24	Practice	Positive adjective	Negative adjective + “street vendor”
4	72	Test	Positive adjective	Negative adjective + “street vendor”

When the terms (e.g., vendors, small traders) and the adjective items (e.g., Good-mannered, Stingy) belong to the same category, participants were asked to press the “F” key for reaction; when they do not belong to the same category, press the “J” key for reaction.

### The Reliability and Validity of “SC-IAT”

We calculated the internal consistency coefficient in this study according to the calculation method of the internal consistency coefficient of the “SC-IAT” proposed by Karpinski and Steinman (2006). That is, we divided the data of each subject into two parts according to odd and even numbers, calculated the average response time of compatible tasks and incompatible tasks, and found the difference between the two. In this way, each subject will get two differences, i.e., an odd difference and an even difference. Then, the correlation coefficients of all subjects were calculated with odd and even differences. After Spearman–Brown correction, the internal consistency coefficient is 0.78, i.e., greater than 0.7, which indicates that the test is reliable.

## Results

In consolidating the data, we gave an additional 400 ms to participants who answered incorrectly. The data with an error rate >20% were removed. In order to avoid the influence of extreme outliers, the data with a response time of <350 ms and >2,000 ms were also removed. After the above-mentioned data processing, we first averaged the response time data (Table 3).

**Table 3:** Means and SDs of SC-IAT after reading positive and negative posts (*N* = 240).

Groups (reading time, min)	Street vendors + positive		Street vendors + negative		*df*	*t*	*p*
	*M*	*SD*	*M*	*SD*			
35	771.15	128.62	820.43	125.42	29	9.87	<0.001
25	823.58	244.32	880.24	237.69	29	6.31	<0.001
15	829.33	210.16	883.69	235.17	29	5.96	<0.001
5	875.12	132.58	846.39	105.22	29	−3.37	0.016

The *t*-test results revealed that when participants had more time to read posts (i.e., the overload was low), their reaction times differed in the two phases. They were more likely to associate “street vendors” with positive adjectives and hold a positive attitude toward them. However, when the reading time was 5 min (i.e., the overload was high), their reaction times did not differ in the two phases (*t* = −3.37, *p* = 0.016), they were not sure about their previous attitude, and the information overload made them confused.

**Figure 1:**
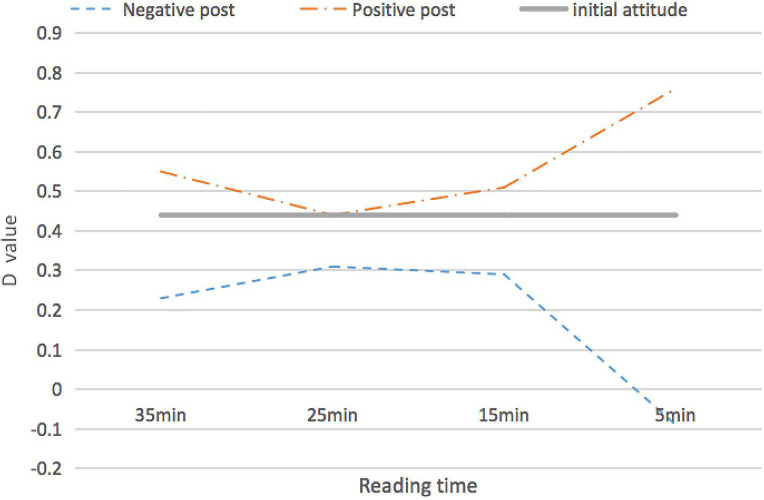
D-values of Single Category Implicit Attitude Test (SC-IAT) under different reading times.

Then, we calculated the D-values of the participants, i.e., to average the difference of the mean difference of the response time in different phases of the experiment. The results were taken as references for the subsequent analysis of implicit attitudes. Specifically, the mean response time of the control group was subtracted from that of the baseline group in the formal experiments. Therefore, in the SC-IAT experiment, a negative D-value indicated that the attitude of the participant toward “street vendors” was negative. At the same time, the greater the absolute D-value, the more evident the deviation of the attitude. The final statistics are shown in Table 4 and Figure 1.

**Table 4:** D-values of SC-IAT after reading posts (*N* = 240).

Posts	Reading time (min)	*M*	*SD*
Negative post	35	0.23	0.04
	25	0.31	0.05
	15	0.29	0.07
	5	−0.09	0.04
Positive post	35	0.55	0.05
	25	0.44	0.09
	15	0.51	0.03
	5	0.76	0.10

Before reading posts, the D-value was 0.44. Furthermore, one-way ANOVA on D-values after reading negative posts indicated the main effect of reading time. There was a significant main effect of reading time, *F*(3,116) = 3.44, *p* = 0.012, and *η*^2^ = 0.05. Of note, 35 and 5 min [*M_(I-J)_* = 0.32, *SD* = 0.02, *p* = 0.021], 25 and 5 min [*M_(I-J)_* = 0.40, *SD* = 0.01, *p* = 0.002], and 15 and 5 min [*M_(I-J)_* = 0.38, *SD* = 0.01, *p* = 0.008] reading time groups had different attitude change toward “street vendors.” Similarly, we also found that reading time had different effects on attitude change in positive posts condition, *F*(3,116) = 3.02, *p* = 0.031, *η*^2^ = 0.03, 35 and 5 min [*M_(I-J)_* = −0.21, *SD* = 0.02, *p* = 0.049], 25 and 5 min [*M_(I-J)_* = −0.32, *SD* = 0.01, *p* = 0.006], and 15 and 5 min [*M_(I-J)_* = −0.25, *SD* = 0.01, *p* = 0.026]. The influence of overload is not a simple linear tendency. The attitude of users first went down when they got more overload, and then it changed strongly with the direction of the post.

**Figure 2:**
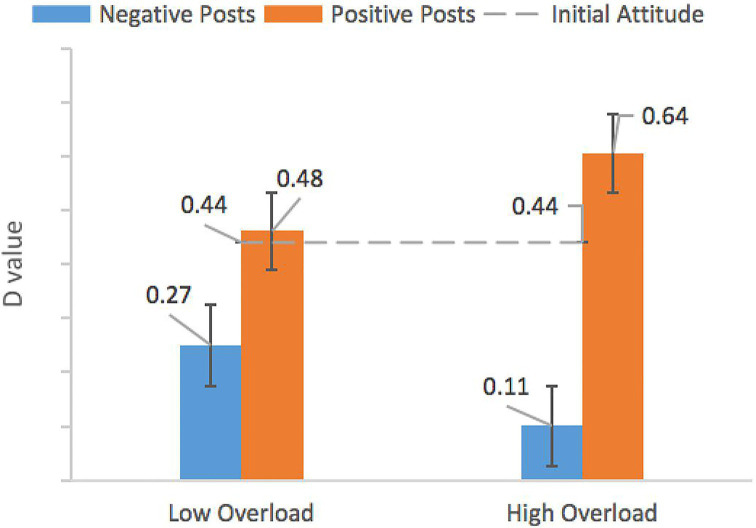
D-values of SC-IAT under two information overload levels. Error bars represent ±SE.

Furthermore, we conducted a 2 (post-attitude: positive vs. negative) × 2 (reading time: low overload vs. high overload) ANOVA. We combined 35 and 25 min reading time group into low overload group; meanwhile, we combined 15 and 5 min reading time group into high overload group, as shown in Figure 2. There was a significant effect of posts attitude, *F*(3,236) = 4.65, *p* < 0.001, *η*^2^ = 0.12. Relative to those read positive posts (*M* = 0.55, *SD* = 0.07), participants had a stronger attitude change after reading negative posts (*M* = 0.19, *SD* = 0.06). The main effect of reading time was not significant, *F*(3,236) = 0.06, *p* = 0.942, *η*^2^ = 0.01. Critically, the two-way interaction effect was significant, *F*(3,236) = 9.02, *p* = 0.024, *η*^2^ = 0.06. After reading negative posts, participants under high information overload (*M* = 0.11, *SD* = 0.07) had a stronger attitude change and their attitude toward the “street vendors” worse than low information overload condition (*M* = 0.27, *SD* = 0.03). Similarly, after reading positive posts, participants under high information overload (*M* = 0.64, *SD* = 0.05) had a stronger attitude change, and positive posts make their attitude toward the “street vendors” better than low information overload condition (*M* = 0.48, *SD* = 0.06). The initial attitudes of participants toward vendors have changed significantly after reading posts under high overload level. The information overload undermines the attitude change defense mechanism of the participants, and users were more easily to be influenced.

## Discussion

Weibo and other social network sites are becoming the salient components of communication platforms and even a public opinion battlefield. In this study, the IAT explains how social media easily change the attitude of users toward a certain group. Furthermore, the information overload, which is brought by the uncertainty, complexity, and other characteristics of social media, could finally destroy the attitude defense of users and make the voice stronger.

Specifically, when the information overload is low, it will be easier to persuade with a lot of repetition. Similar to other studies, people are persuaded by aggressive information leading to changes in their attitude (Bless and Forgas, 2000). The significant effects of the information overload in our study were consistent with the findings by Qiu-mei (2015), which indicates that information overload had directed effects on the attitude change of street vendors. As the voice becomes louder, when the information is repeated many times, a classic overexposed effect arises (McCullough and Ostrom, 1974). The information is presented frequently, thereby leading to a less positive evaluation of the information. The presentation of the information multiple times causes a reduction in the effect of persuasion. This is attributed to individual defenses because people feel subjected to pressure from excessive persuasion (Briñol et al., 2004). In most cases, people refuse to change their thinking and evaluation. Cognizant to this, they actively seek ways to make the existing evidence not change the consistency of their attitude when they encounter information that is inconsistent with their own attitude. They do so by interpreting ambiguous evidence in a manner consistent with their attitude such as actively seeking opinions and evidence consistent with their own attitude to reduce cognitive dissonance (Janssen et al., 2010). Users have an attitude defense because of the cognitive load caused by the characteristics of social media. However, after a short defense, participants are still unable to find more evidence and comprehension to support their initial views. Their perspective finally changed same with posts voice under the influence of several consistent and reliable information. There is a contribution in this research. Under the environment of information overload, the attitude change of people is not a simple linear, and there is a resistance to the information process in the outside world. Although the positive media information could make readers view the problems in a positive way, in fact, people added rational judgment in this process, which suggests that such positive information could not directly lead to positive feelings, and vice versa (see Figure 1).

Nevertheless, this study was limited by several factors. This research only discusses the attitude changes that occur when users see positive posts or negative posts. However, in reality, the actual posts on social media are often more complicated, which means that it is difficult to be just one-sided. In addition, this study is about the published post, and how the comments below the post affect should be further explored. Moreover, whether the status of the publishing account is an authoritative account or a personal account would affect their attention interpretation of information should also be further explored. In addition, the information on social media can evoke the kinds of emotions of people (including positive and negative emotions) and affect the attitudes and behaviors of people (Poggi and D’Errico, 2010; Hudson et al., 2015), but we did not control the effect of emotion on the attitude change of people, and we will solve the problems in the future study.

## Conclusion

Evidently, during a public opinion storm on social media, the attitude of users is not directly changed, but is a wave that shows obedience first, and resistance but acceptance totally in the end. The wrong amount of posts in the storm would not popularize the information but do a disservice. Furthermore, the cognitive overload plays an important role in persuasion on social media. Adding amounts or decorating content, which could increase cognitive load, may contribute to convince others. Apparently, there are several different stages during the persuasion process. Thus, identifying these stages and using the proper strategy would be more effective.

## Data Availability Statement

The raw data supporting the conclusions of this article will be made available by the authors, without undue reservation.

## Ethics Statement

The studies involving human participants were reviewed and approved by Ethics Committee of the Department of Psychology Peking University. The patients/participants provided their written informed consent to participate in this study.

## Author Contributions

YW: conceptualization, investigation, and writing – original draft preparation. HL: methodology, software, and data curation. YD: writing – original draft preparation and writing – review and editing. LS: writing – review and editing. All authors contributed to the article and approved the submitted version.

### Conflict of Interest

The authors declare that they have no known competing financial interests or personal relationships that could have appeared to influence the work reported in this paper.
